# Human distribution and spatial-temporal clustering analysis of human brucellosis in China from 2012 to 2016

**DOI:** 10.1186/s40249-020-00754-8

**Published:** 2020-10-13

**Authors:** Pei-Feng Liang, Yuan Zhao, Jian-Hua Zhao, Dong-Feng Pan, Zhong-Qin Guo

**Affiliations:** 1 Department of medical record and statistics, People’s hospital of Ningxia Hui Autonomous region, Yinchuan, 750004 China; 2 Xiangya Medical College, Central South University, Changsha, 410013 China; 3 Department of Epidemiology and Biostatistics, School of Public Health and management, Ningxia Medical University, Yinchuan, 750001 China; 4 Ningxia Center for Diseases Prevention and Control, Yinchuan, 750001 China; 5 Department of Emergency, People’s hospital of Ningxia Hui Autonomous region, Yinchuan, 750004 China

**Keywords:** Human brucellosis, China, Clustering analysis, Geographic information system, SaTScan software

## Abstract

**Background:**

Brucellosis is a major public health issue in China, while its temporal and spatial distribution have not been studied in depth. This study aims to better understand the epidemiology of brucellosis in the mainland of China, by investigating the human, temporal and spatial distribution and clustering characteristics of the disease.

**Methods:**

Human brucellosis data from the mainland of China between 2012 and 2016 were obtained from the China Information System for Disease Control and Prevention. The spatial autocorrelation analysis of ArcGIS10.6 and the spatial-temporal scanning analysis of SaTScan software were used to identify potential changes in the spatial and temporal distribution of human brucellosis in the mainland of China during the study period.

**Results:**

A total of 244 348 human brucellosis cases were reported during the study period of 2012–2016. The average incidence of human brucellosis was higher in the 40–65 age group. The temporal clustering analysis showed that the high incidence of brucellosis occurred between March and July. The spatial clustering analysis showed that the location of brucellosis clustering in the mainland of China remained relatively fixed, mainly concentrated in most parts of northern China. The results of the spatial-temporal clustering analysis showed that Heilongjiang represents a primary clustering area, and the Tibet, Shanxi and Hubei provinces represent three secondary clustering areas.

**Conclusions:**

Human brucellosis remains a widespread challenge, particularly in northern China. The clustering analysis highlights potential high-risk human groups, time frames and areas, which may require special plans and resources to monitor and control the disease.

## Background

Brucellosis is a widespread zoonosis caused by *Brucella* species, which can result in considerable human suffering and huge economic loss in livestock [1–3]. Despite the global annual incidence of half a million cases [4–7], brucellosis had been neglected for a long time. However, with the global economic recovery, the rapid development of cattle and sheep breeding and tourism led to an increase in the spread of brucellosis [8, 9]. Nowadays, brucellosis is prevalent in more than 170 countries around the world, mostly in the Mediterranean, Asia and Central and South America [10–12]. Due to its harmful effects on the agriculture and animal husbandry economy and people’s health, brucellosis is still a serious public health problem.

In China, human brucellosis remains a major public health issue. While the first reported cases were two foreign patients in Shanghai in 1905, which were recorded as Malta fever, several patients in China with similar clinical symptoms had been observed during the 10 years preceding that report. Human brucellosis was reported to be endemic in 25 out of 31 provinces (municipalities and autonomous regions) of the mainland of China [13]. From the 1970s to the early 1990s, the incidence of human brucellosis significantly decreased. However, the situation of brucellosis in China has been largely aggravated in the last 10 years [14]. In 2015, the incidence of the human cases reached 4.18/100 000, rising from the 16th place in 2000 to the 6th place in 2014 in the ranking of statutory infectious diseases. Such a strong upward trend is extremely rare among all reported infectious diseases.

It was previously demonstrated that the global epidemiology of brucellosis has dramatically changed over the past decades, particularly in industrialized countries [15]. The spatial distribution characteristics of a brucellosis outbreak have also distinctly changed [1]. However, further investigation is needed to determine whether this change has temporal and spatial covariance. In this study, we applied a spatial and temporal distribution model on the national surveillance data on human brucellosis to analyze the human and spatial-temporal distribution characteristics and correlations of human brucellosis epidemics in the mainland of China in the period between 2012 and 2016.

## Materials and methods

### Data source

The data of human brucellosis cases from 31 provinces (municipalities and autonomous regions) in the mainland of China between 2012 and 2016 were obtained from the Data Center of China Public Health Science of National population and Health Science Data Sharing Platform in China (URL address: http://www.phsciencedata.cn/Share/index.html?aafa8285-42ae-4dbc-a828-152c2cef6396), which was established in 2004, collecting all the reported cases since the direct network report system of infectious diseases in China. The main contents of the database of human brucellosis include the number of cases, incidence rate, number of deaths and mortality rate in multiple dimensions by the region, age group, gender and occupation. According to the national brucellosis surveillance program in China, this system includes the clinically diagnosed cases and confirmed ones. The clinically diagnosed cases are defined as the presumptive cases (those with both epidemiological history and clinical manifestations), with any positive screening test results. On the other hand, the confirmed cases are defined as the presumptive or clinically diagnosed cases, with any confirmed experimental evidence.

### Statistical analysis

The SPSS software (version 23.0, IBM company, New York, USA) was used for the statistical analysis. Firstly, age clustering was achieved by the hierarchical clustering method, which is a statistical analysis technique that divides the objects into relatively homogeneous groups. According to the characteristics of the objects, they are classified so as to reduce the number of objects. Then, the impact of seasonality on the human brucellosis incidence was explored by performing time series decomposition analysis in the R software (Bell Laboratories, New Jersey, America). A seasonal decomposition was used to break down the monthly time series data into four components: original time series data, trend, seasonality and random effect.

### Global Moran’s *I* value

The Global Moran’s *I* value was used to determine whether there is a global spatial autocorrelation between the provinces. The *I* value ranges from [− 1, 1], such that *I* > 0 indicates a positive spatial correlation, while *I* < 0 indicates a negative spatial correlation; if the *I* value is close to 0, no spatial correlation exists [16]. The larger the absolute value of *I*, the stronger the correlation. When *|Z|* > 1.96, *P* < 0.05 was considered to be statistically significant and a spatial autocorrelation existed [17, 18]. The global spatial autocorrelation was conducted using the spatial autocorrelation tool in the ArcGIS software (Version, 10.6, Environmental Systems Research Institute, Redlands, USA).

### Space-time scan

The SaTScan software (developed by Martin Kulldorff together with Information Management Services Inc., America), developed by Kuldlorff based on the spatial dynamic window scanning statistics, was used to explore the spatial, temporal and spatial-temporal clustering of human brucellosis. Besides, it was used to verify whether the time and geographic clustering of human brucellosis was caused by random variation or not [19]. By calculating the likelihood ratio of the spatial unit attributes within and outside the dynamic window area under different centers and radiuses, SaTScan makes the statistical inference and explores the maximum possible clustering area using Monte Carlo simulation for the statistical significance evaluation [20]. For each possible spatial-temporal clustering area, when *P* < 0.05, the larger the log-likelihood ratio (LLR) value, the more the likelihood that the area covered by the scanning dynamic window represents the clustering area [21]. Finally, the window with the largest LLR value is selected as the maximum possible clustering area, while other windows with statistical significance represent the secondary possible clustering area. The high spatial clustering areas of brucellosis in the mainland of China were analyzed using a retrospective spatial analysis method and a Poisson distribution model.

Selecting the maximum radius of the scanning window and the maximum length of the temporal scanning window was very important, since the results of the spatial-temporal scan are sensitive to these parameters [22]. The default size settings for the window and time are usually set to 50%, although some studies have questioned whether this is appropriate [23]. If the window size is too large, a high false positive rate may occur. Similarly, a high false negative rate may occur if the window size is too small [24]. The appropriate selection of the scan window size has been explored by many studies, which mainly showed that the number of areas covered by a single clustering area should not exceed 15% of the number of overlapping areas [25, 26]. In addition, some studies also used this standard in regular scan statistics [25]; thus, we used this experience as a reference in our study. Finally, we selected the spatial window covering 30% of the population at risk of the total data in the study period to perform our research.

Three data banks were connected to the program for the analysis: one with the latitudes and longitudes of the centroids of each province, another with the populations of the provinces by the year and a third with the number of cases per province and by the year [27].

## Results

### Descriptive analysis

A total of 244 348 human brucellosis cases were reported from 31 provinces during the study period. Among all the represented years, there are the most number of cases (57 222) in 2014, except for the Tibet Autonomous Region. There was a small increase in the incidence during the study period (2.9328/100 000 in 2012 to 3.4388/100 000 in 2016).

### Clustering analysis

#### Human clustering analysis

At present, the incidence of brucellosis is gradually expanding to include all age groups in addition to the previously affected young and middle-aged population. During the period of 2012–2016, the average incidence of human brucellosis was higher in the age group of 40–65 years. On the other hand, the incidence of brucellosis in other age groups was relatively low, and the age groups under 20 and over 80 had a much lower incidence. The concrete incidence distribution of each age group is shown in Table 1.

**Table 1:** Age distribution of human brucellosis incidence in the mainland of China from 2012 to 2016

Age group	2012	2013	2014	2015	2016
0–4	1.63	1.73	3.02	3.63	2.73
5–9	1.80	2.28	3.25	3.98	2.78
10–14	0.40	0.50	0.85	1.00	0.77
15–19	0.67	0.66	0.97	0.95	0.80
20–24	1.25	1.19	1.39	1.28	1.05
25–29	2.50	2.55	3.27	3.25	2.81
30–34	3.53	3.55	4.68	4.49	3.71
35–39	3.46	3.42	4.53	4.19	3.51
40–45	4.64	4.77	6.13	5.59	4.47
45–49	4.81	5.34	6.14	6.18	5.18
50–54	6.18	7.29	9.89	10.33	8.98
55–59	5.33	6.12	7.71	7.03	5.71
60–64	5.27	6.30	8.55	8.86	7.27
65–69	3.45	4.48	6.73	7.51	5.69
70–74	1.93	2.45	3.55	3.91	3.05
75–79	1.39	1.69	1.86	1.94	1.64
80–84	0.74	0.86	1.09	1.25	0.87
> 85	0.41	0.86	0.46	0.69	0.44

**Fig. 1:**
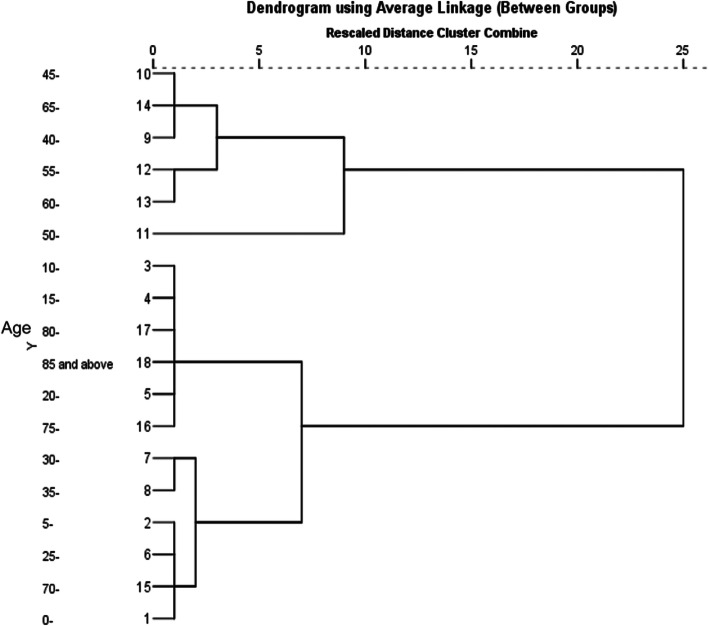
Age cluster distribution of the human brucellosis incidence in the mainland of China from 2012 to 2016 (The average incidence of human brucellosis among different age groups during 2012 to 2016 was divided into three clusters, with the highest incidence in the 40–65 groups)

As shown in Fig. 1, the results of the hierarchical clustering show that the average incidence of human brucellosis among different age groups during the period of 2012 to 2016 was divided into three clusters. The highest incidence was in the cluster of the 40–65 groups. The second cluster included the groups of 10–14, 15–19, 80 and above, 20–24 and 70–74. Finally, the incidence rates of the 30–34, 35–39, 5–9, 25–24, 70–74 and 0–4 groups were clustered into another cluster.

#### Temporal clustering analysis

**Fig. 2:**
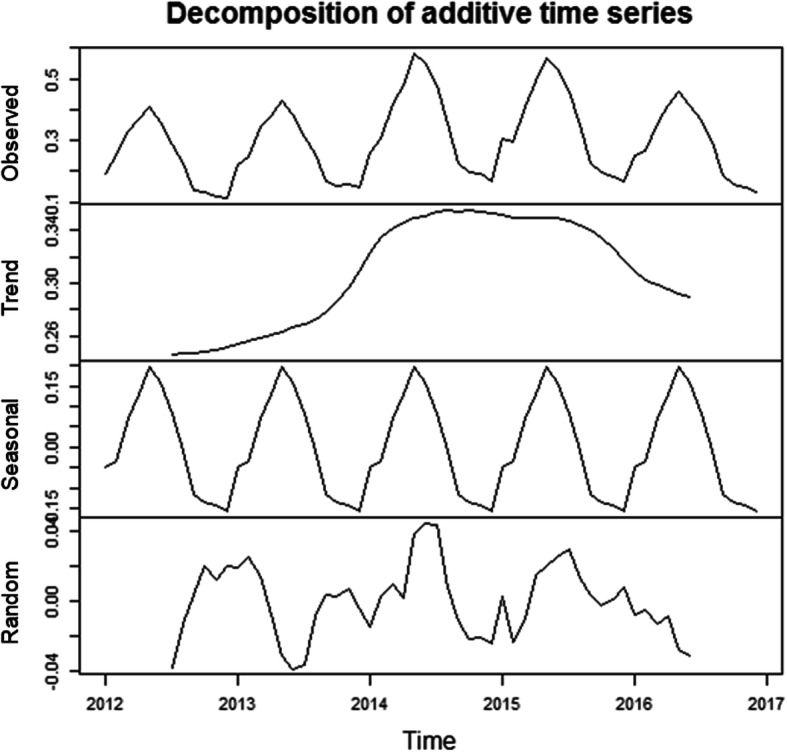
The seasonal distribution of monthly human brucellosis cases in the mainland of China from 2012 to 2016 (The seasonal decomposition of the brucellosis incidence showed that there was an increasing trend of human brucellosis with distinct seasonality)

The results of the seasonal decomposition of the brucellosis incidence showed strong seasonal characteristics, as shown in Fig. 2. Similarly, the temporal cluster analysis showed a consistent result, with a high incidence of brucellosis occurring between March and July annually (Table 2). The time frame with a high clustering for human brucellosis in the whole study period was observed from January 2014 to December 2015. During this period, a total of 113 111 human brucellosis cases were reported, and the risk of human brucellosis-related events was 31% (relative risk [RR] = 1.31, *P* = 0.001) higher than the risk during other periods. In addition, the overall brucellosis incidence increased during the study period, but slightly declined in 2016.

**Table 2:** Temporal clustering of reported brucellosis cases in the mainland of China from 2012 to 2016

Year	Cluster time frame	Observed*	Expected*	RR	LLR	*P*
2012	2012/2 to 2012/7	27 075	19 649.54	2.20	2856.35	0.001
2013	2013/2 to 2013/7	28 463	21 564.29	1.93	2223.25	0.001
2014	2014/3 to 2014/8	38 903	28 291.64	2.22	4123.72	0.001
2015	2015/3 to 2015/7	33 492	23 888.54	1.98	3265.39	0.001
2016	2016/3 to 2016/8	31 495	23 698.30	1.99	2631.76	0.001
2012–2016	2014/1 to 2015/12	113 111	97 193.89	1.31	2138.16	0.001

Observed*: Number of Observed cases in a cluster

Expected*: Number of Expected cases in a cluster

*RR* Relative risk, *LRR* Log-likelihood ratio

#### Spatial clustering analysis

We obtained the Moran’s *I* value, variance, *Z* score and *P* value from 2012 to 2016 using provincial units to carry out the global autocorrelation analysis (see Table 3). The values of Moran’s *I* were 0.1179 and 0.1181 for 2013 and 2014, respectively, while the *Z* values were greater than 1.96 (all *P* < 0.05), which indicates that the incidence of brucellosis in China between 2013 to 2014 had a non-random distribution; thus, further spatial clustering analysis of human brucellosis was needed.

**Table 3:** Global auto-correlation Moran’s *I* value of brucellosis in the mainland of China from 2012 to 2016

Year	Moran’s *I*	Variance	*Z* Score	*P* Value	Aggregation
2012	0.076004	0.003579	1.827598	0.067610	NO
2013	0.117938	0.004963	2.147162	0.031780	YES
2014	0.118131	0.005379	2.065198	0.038904	YES
2015	0.093595	0.005191	1.761692	0.078121	NO
2016	0.092068	0.005165	1.744833	0.081014	NO

**Fig. 3:**
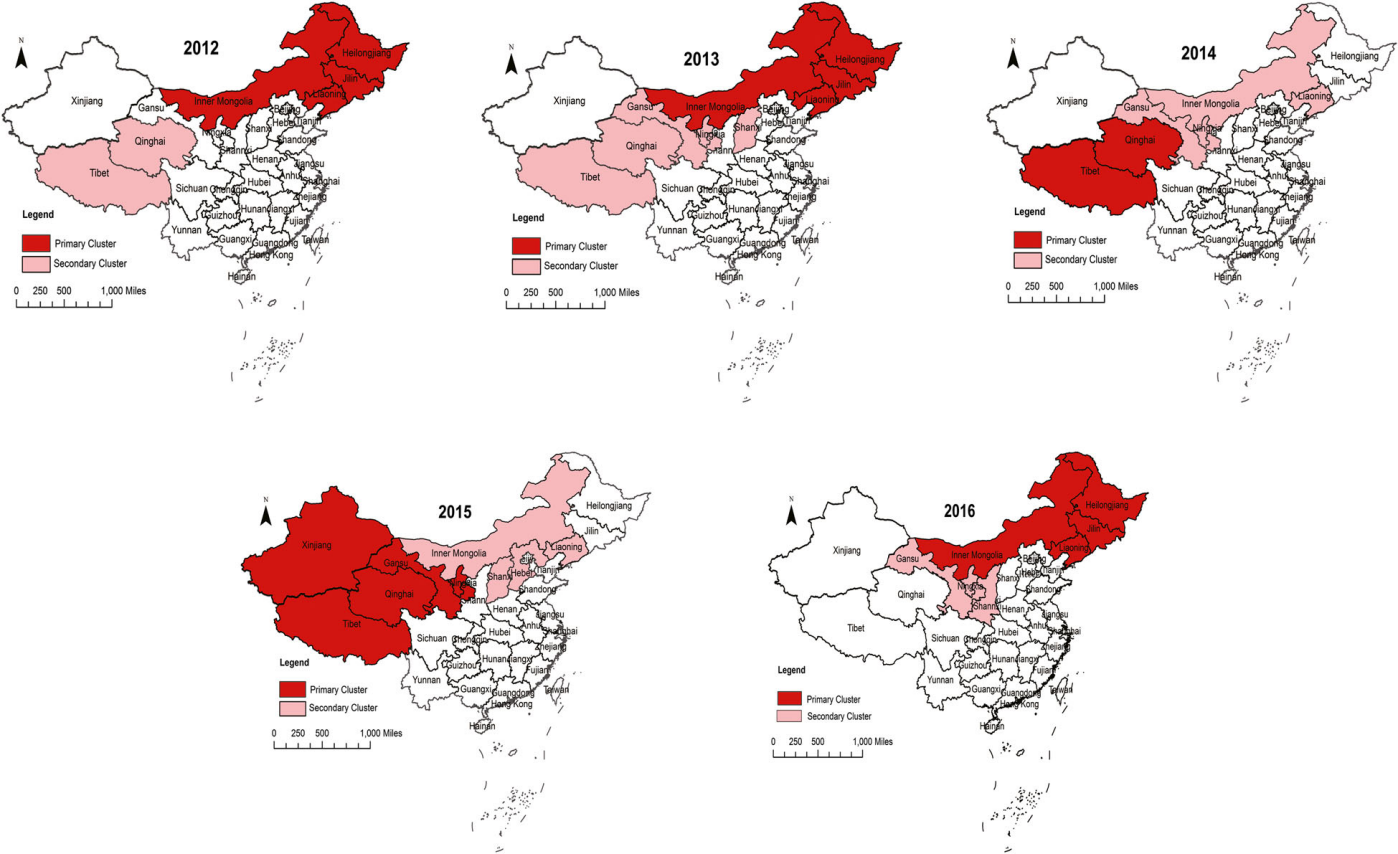
Spatial clustering of the reported brucellosis cases in the mainland of China from 2012 to 2016 (The darker color represents the primary clustering area, and the lighter color represents the secondary clustering area. It is obvious that high incidence areas were concentrated in the north of China. [Color is required for this figure])

As shown in Fig. 3, the spatial clustering analysis of the incidence of human brucellosis from 2012 to 2016 showed that the location of the brucellosis clustering in the mainland of China remained relatively stationary, mainly concentrated in most parts of northern China. From 2012 to 2015, one region was located in the northeast of China’s mainland, and included Heilongjiang, Jilin, Liaoning and Inner Mongolia, while the other region contained Tibet, Xinjiang, Qinghai, Ningxia, Gansu and Shanxi. In 2016, there were relatively few clustering regions, such that the primary clustering area included Heilongjiang, Jilin, Liaoning and Inner Mongolia, and the secondary clustering area included Gansu, Ningxia and Shanxi.

#### Spatial-temporal clustering analysis

**Fig. 4:**
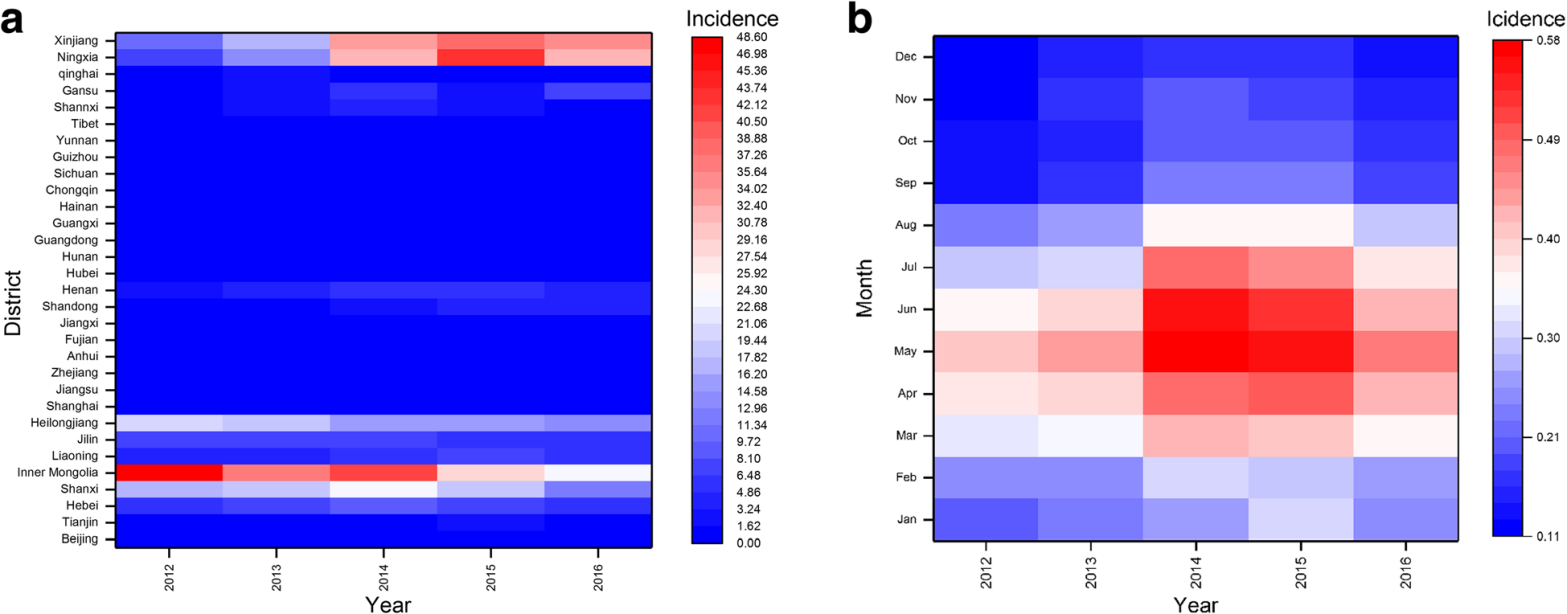
Spatial-Temporal clustering of the reported brucellosis cases in the mainland of China from 2012 to 2016. (**a** The distribution of human brucellosis incidence in different provinces of China from 2012 to 2016, **b** The distribution of human brucellosis incidence in each month from 2012 to 2016. Red represents a high incidence, while blue represents a low incidence. [Color is required for this figure])

A heat map was drawn for the regions and time periods of the human brucellosis incidence. It was found that the areas of Xinjiang, Ningxia, Heilongjiang, Inner Mongolia and Shanxi had a high incidence during the study period (Fig. 4a), which was much higher than that in other regions for the same period. At the same time, brucellosis tends to occur from March to August, with the highest incidence in May, particularly in 2014 and 2015 (Fig. 4b).

**Fig. 5:**
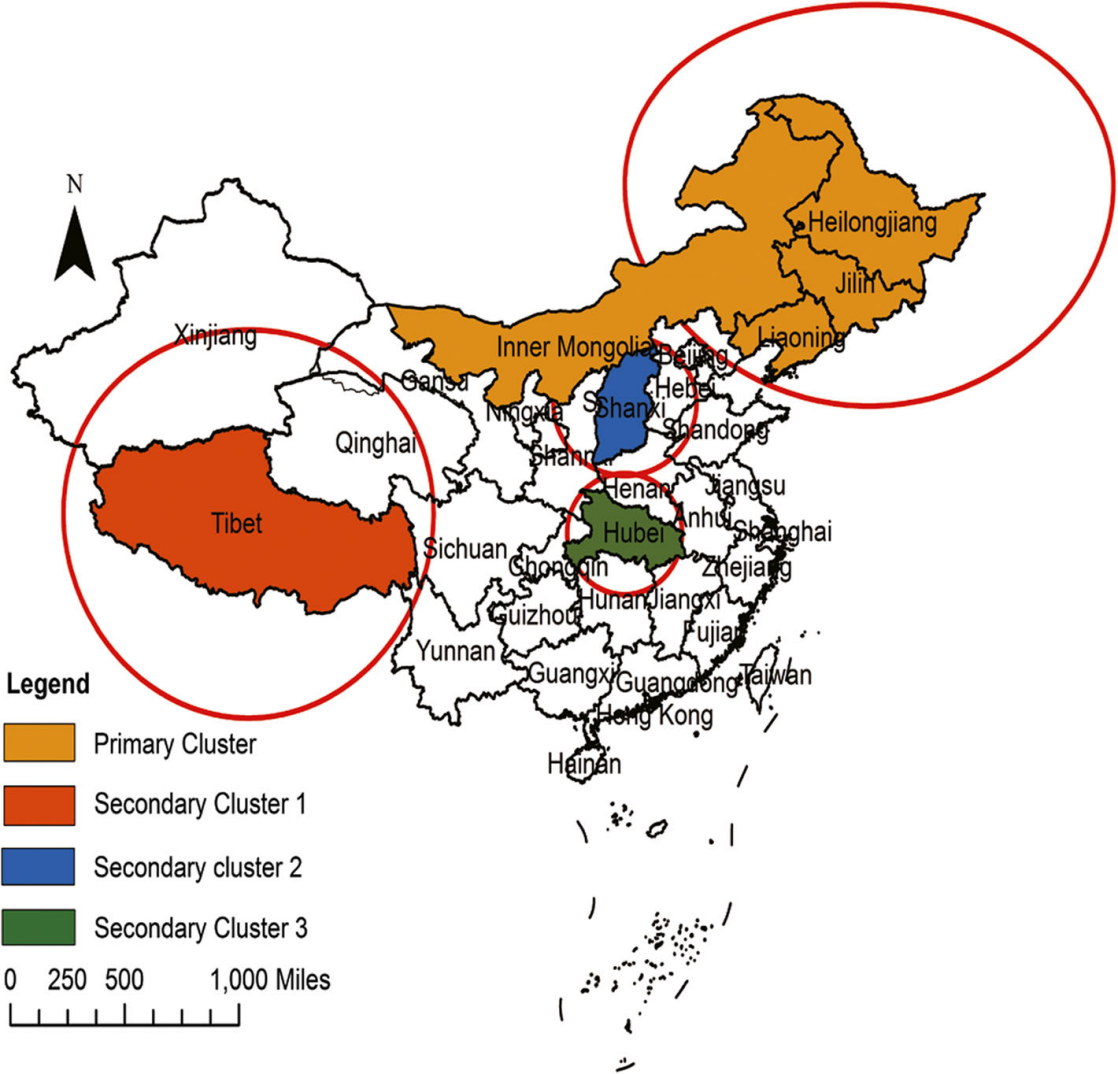
Spatial-Temporal clustering of the reported brucellosis cases in the mainland of China from 2012 to 2016. (The circles represent the clustering areas, such that the largest represents the primary clustering area, and the remaining ones represent the secondary clustering areas. (Color is required for this figure))

Finally, we identified both spatial and temporal clusters of high incidence districts per zone. As shown in Table 4 and Fig. 5, the results of the spatial-temporal cluster analysis for the reported human brucellosis cases in 31 provinces of the mainland of China from 2012 to 2016 included one primary clustering area and three secondary clustering areas. The primary clustering area was located in the northeast of China, including Inner Mongolia, Heilongjiang, Jilin and Liaoning, and the high-risk time frame was from January 2012 to December 2013 (RR = 5.17, LLR = 33 228.98, *P* < 0.001). The three secondary clusters were mainly distributed in the northeast of China with several relatively small areas in central China, and the cluster time frames mainly ranged from January 2014 to December 2016.

**Table 4:** Spatial-temporal clustering of reported human brucellosis in the mainland of China from 2012 to 2016

Cluster type	Coordinates/ radius (km)	Period	N*	Observed*	Expected*	RR	LLR	*P*
Primary Cluster	Heilongjiang (47.86 N,127.76E) /1146.37	2012/1/1–2013/12/31	4	42 951	9673.18	5.17	33 228.98	< 0.001
Secondary Cluster 1	Tibet (31.49 N, 88.44E) /1109.00	2015/1/1–2016/12/31	3	17 264	2364.70	7.78	19 889.42	< 0.001
Secondary Cluster 2	Shanxi (37.57 N,112.29E)/ 399.03	2014/1/1–2015/12/31	2	27 582	7932.50	3.79	15 563.23	< 0.001
Secondary Cluster 3	Hubei (30.98 N,112.27E)/ 346.76	2015/1/1–2015/12/31	2	5830	5498.39	1.06	10.03	< 0.001

N*: number of Counties in a cluster

Observed*: Number of Observed cases in a cluster

Expected*: Number of Expected cases in a cluster

*RR* Relative risk, LRR* Log-likelihood ratio

## Discussion

Due to its resurgence in China and abroad, brucellosis is still regarded as a serious public health problem, and the importance of restarting the control strategy cannot be overemphasized. Describing the human, spatial and temporal cluster distribution of human brucellosis represents a corner stone to prevent and eliminate brucellosis. This work provides the theoretical support for the prevention and control of human brucellosis in the context of local conditions.

Despite the fact that there is no age preference in brucellosis and all people can be infected, our findings still show a clear age difference. The incidence of brucellosis in people aged 25 years is significantly higher than that in those aged less than 25 years, and the incidence in people aged more than 65 old is also lower. It is not difficult to see that the distribution of age is based on whether it is an important family labor force. In rural Chinese families, most middle-aged and elderly people aged between 40 and 65 are raising livestock, so the incidence of brucellosis among this age group is the highest. The presence in people from other age groups may be related to drinking unpasteurized milk [28, 29].

It is well known that seasonal identification is a key step in the brucellosis prevention strategy. The results of our temporal cluster analysis show that there is a distinct seasonality in human brucellosis. The main cluster time of the reported cases is from March to July, accounting for 58.86% of the total cases from 2012 to 2016. The reason might be that these months represent the time of lambing in the agricultural and pastoral areas of northern China. It may also be due to warm temperatures suitable for the transmission of zoonosis, with similar seasonal characteristics in other countries [5, 30–33]. At the same time, according to some reported research findings, the factors of temperature, sunshine, wind speed, altitude and rainfall will affect the introduction of brucellosis [34–36].

The results of the spatial clustering analysis of the human brucellosis incidence in 31 provinces in the mainland of China showed that the spatial cluster existed in every year from 2012 to 2016. The distribution of the clustering regions was similar for each year, which meant that the areas with a high incidence of brucellosis in China are concentrated in the northern animal husbandry areas and their adjacent areas; this was in accordance with the conclusion reported by other studies that Ningxia, Xinjiang, Qinghai and Inner Mongolia belong to the four major pastoral areas in China [14]. Meanwhile, Heilongjiang, Jilin, Liaoning, Gansu and Shanxi provinces also belong to the cluster zone, possibly due to their proximity to the regions with a high incidence of brucellosis and the close exchange of livestock and meat. At the same time, there is no doubt that most of the areas with a high brucellosis incidence are China’s economically underdeveloped areas, due to their poor economy, high proportion of minorities and underdeveloped medical level. The government and individuals invested less in public health, so they have not performed well in timely quarantine and immunization, which led to a serious condition of brucellosis.

The spatial-temporal cluster analysis identified one primary cluster and three secondary clusters of human brucellosis incidence. The primary cluster was located in the areas of Heilongjiang, Jilin, Liaoning and Inner Mongolia, and the clustering time was concentrated from January 2012 to December 2013, which indicated that the prevention and control measures in these regions still need to be strengthened, not only in the distant past, but also in recent years. Other secondary clusters were distributed in northwestern China and scattered in central China mainly from January 2014 to December 2016. This means that brucellosis moves from north to south; thus, prevention and control awareness should also be established in these areas.

Our study had some limitations. Firstly, the incidence of human brucellosis was underestimated to some extent, as our data were passively collected by a monitoring system, while the quality of the surveillance data was influenced by comprehensive factors, such as the capacity of local health workers, availability of laboratory diagnostics, level of awareness about the need to visit doctors and so on, all of which may have affected the accuracy of the results of our study. Secondly, the method of spatial-temporal scan statistics that we used to detect clusters in different space and time periods relies only on circular space scans and cylindrical spatial-temporal scans and does not consider irregular spaces.

Given the development in animal husbandry in China, there is an urgent need to strengthen the prevention and detection of brucellosis. This spatial-temporal clustering study of brucellosis helps to identify high-risk areas and time frames for brucellosis, and provides a basis for the decision-making of relevant departments to a certain extent. In terms of the distribution characteristics of the brucellosis epidemic in this study, we suggest to further strengthen the detection of brucellosis in northern China and keep on the monitoring in central China. In addition, effective methods for controlling brucellosis should be applied in southern China, where the incidence of brucellosis is relatively low. Governments at all levels should work on establishing a joint prevention and control mechanism among high-incidence areas. Relevant departments in different regions should strengthen both the prevention and control throughout the year, while appropriately increasing the resources in these areas to curb the spread of brucellosis.

## Conclusions

It may be concluded that human brucellosis continues to be a widespread challenge in the mainland of China, especially in northwestern provinces, which represent high-risk areas. Further research should focus on analyzing the environmental, humanistic and socioeconomic factors in order to determine the risk factors affecting the occurrence and transmission of brucellosis. Such information has the potential to provide critical guidelines for policy makers to initiate appropriate prevention measures and control strategies, aimed at susceptible areas that might be high-risk areas, and to prevent or reduce the incidence of human brucellosis in these areas.

## Data Availability

The datasets analyzed during the current study are available from the corresponding author on reasonable request. The URL link of the dataset is: http://www.phsciencedata.cn/Share/ky_sjml.jsp?id=aafa8285-42ae-4dbc-a828-152c2cef6396

## Abbreviations

CDC: Centers for Disease Control and Prevention
WHO: World Health Organization;
GIS: Geographic Information System
LLR: Likelihood Rate
RR: Relative Risk
